# VEGF dose controls the coupling of angiogenesis and osteogenesis in engineered bone

**DOI:** 10.1038/s41536-023-00288-1

**Published:** 2023-03-13

**Authors:** Andrea Grosso, Alexander Lunger, Maximilian G. Burger, Priscilla S. Briquez, Francesca Mai, Jeffrey A. Hubbell, Dirk J. Schaefer, Andrea Banfi, Nunzia Di Maggio

**Affiliations:** 1 Regenerative Angiogenesis Laboratory, Department of Biomedicine, Basel University Hospital and University of Basel, Hebelstrasse 20, 4031 Basel, Switzerland; 2 Department of Plastic, Reconstructive, Aesthetic and Hand Surgery, Basel University Hospital, Petersgraben 4, 4031 Basel, Switzerland; 3 Pritzker School of Molecular Engineering, University of Chicago, 5640 S Ellis Ave, Chicago, IL 60637 USA; 4 Department of General and Visceral Surgery, Medical Center – University of Freiburg, Faculty of Medicine, University of Freiburg, 79106 Freiburg, Germany

**Keywords:** Mesenchymal stem cells, Regenerative medicine

## Abstract

Vascular endothelial growth factor-A (VEGF) physiologically regulates both angiogenesis and osteogenesis, but its application in bone tissue engineering led to contradictory outcomes. A poorly understood aspect is how VEGF dose impacts the coordination between these two processes. Taking advantage of a unique and highly tunable platform, here we dissected the effects of VEGF dose over a 1,000-fold range in the context of tissue-engineered osteogenic grafts. We found that osteo-angiogenic coupling is exquisitely dependent on VEGF dose and that only a tightly defined dose range could stimulate both vascular invasion and osteogenic commitment of progenitors, with significant improvement in bone formation. Further, VEGF dose regulated Notch1 activation and the induction of a specific pro-osteogenic endothelial phenotype, independently of the promotion of vascular invasion. Therefore, in a therapeutic perspective, fine-tuning of VEGF dose in the signaling microenvironment is key to ensure physiological coupling of accelerated vascular invasion and improved bone formation.

## Introduction

Large bone defects due to trauma, surgery or other pathological conditions cannot be repaired by spontaneous regeneration^1^ and their treatment is an unmet challenge in clinical practice^2^. The use of tissue-engineered bone grafts is promising for the repair of clinical-size defects but has yet to make a significant impact for patients. One of the critical issues to be solved towards this goal is the need to coordinate the formation of a vascular supply within the engineered grafts to form adequate amounts of physiological bone tissue. The role of the vasculature in bone formation is two-fold: 1) the supply of oxygen and nutrients, which requires a rapid establishment of perfusion^3^, and 2) the exchange of a complex crosstalk of paracrine signals that coordinate bone formation, as well as osteoprogenitor differentiation and behaviour, i.e. a so-called angiocrine function^4–7^. Therefore, under physiological conditions, the bone repair is a rapid, well-orchestrated and efficient process that involves a tight coupling of osteogenesis and angiogenesis.

An attractive strategy to drive vascular growth into osteogenic grafts is the supply of specific signals that regulate physiological angiogenesis. Vascular endothelial growth factor-A (VEGF) is the master regulator of vascular growth both in normal and pathological angiogenesis and is therefore the target for inducing the therapeutic growth of new blood vessels^8^. However, simple provision of VEGF to osteogenic grafts has been met with limited success, due in part to the inherent complexity of the biological connections between angiogenesis and osteogenesis^9–13^. There is therefore a need to better elucidate how VEGF regulates the coupling of the two processes, in order to exploit its biological potential to drive bone vascularization.

One key aspect that needs to be precisely understood is how VEGF dose impacts the coordination between vascular growth and bone formation. Several lines of evidence indicate that VEGF levels of expression must be carefully controlled during osteogenesis in vivo. In fact, while physiological levels are required to maintain bone homeostasis, loss of VEGF can impair osteoblast differentiation and bone deposition^14,15^, whereas over-expression can stimulate excessive bone resorption and likewise lead to bone loss^3^. However, very little is known about how VEGF dose regulates the therapeutic regeneration of vascularized bone, due to the difficulty of precisely controlling the spatiotemporal distribution of VEGF available in osteogenic grafts (reviewed by Martino et al.^16^). In this regard, it should be also considered that: a) the physiological presentation of VEGF to its target cells requires the interaction with extracellular matrix (ECM), which orchestrates its activity by regulating its local concentration, bioavailability and signalling^17^, and b) tissue regeneration after damage starts in all cases with the deposition of a provisional fibrin-based matrix derived from blood clotting and fibrin is also widely used for tissue engineering approaches^18^. Therefore, we took advantage of a protein engineering approach to decorate fibrin matrices with tunable and homogeneous concentrations of VEGF and recapitulate its physiological matrix-bound presentation^19,20^. An engineered version of mouse VEGF_164_ was fused to the octapeptide substrate sequence for the transglutaminase coagulation Factor XIII (TG-VEGF), whereby upon cross-linking of fibrinogen monomers into a fibrin hydrogel, TG-VEGF is covalently linked to the fibrin network^21–23^. Thus, TG-VEGF is presented to embedded osteoprogenitors and invading endothelial cells in the context of ECM, allowing the engineering of a specific signalling microenvironment. Here we investigated whether and how VEGF dose regulates the effective coupling of angiogenesis and osteogenesis for the therapeutic regeneration of vascularized bone.

## Results

### Increasing VEGF dose delays vascular invasion and determines the distribution of vascular growth

**Fig. 1:**
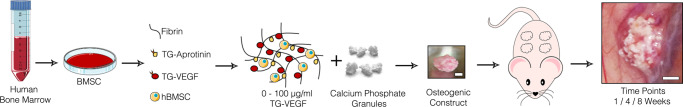
Study design for the ectopic model of bone formation. Osteogenic constructs were generated by combining in vitro expanded human BMSC with calcium phosphate granules in a fibrin hydrogel decorated with different doses of TG-VEGF, ranging from 0 to 100 µg/ml; scale bars = 2 mm.

**Fig. 2:**
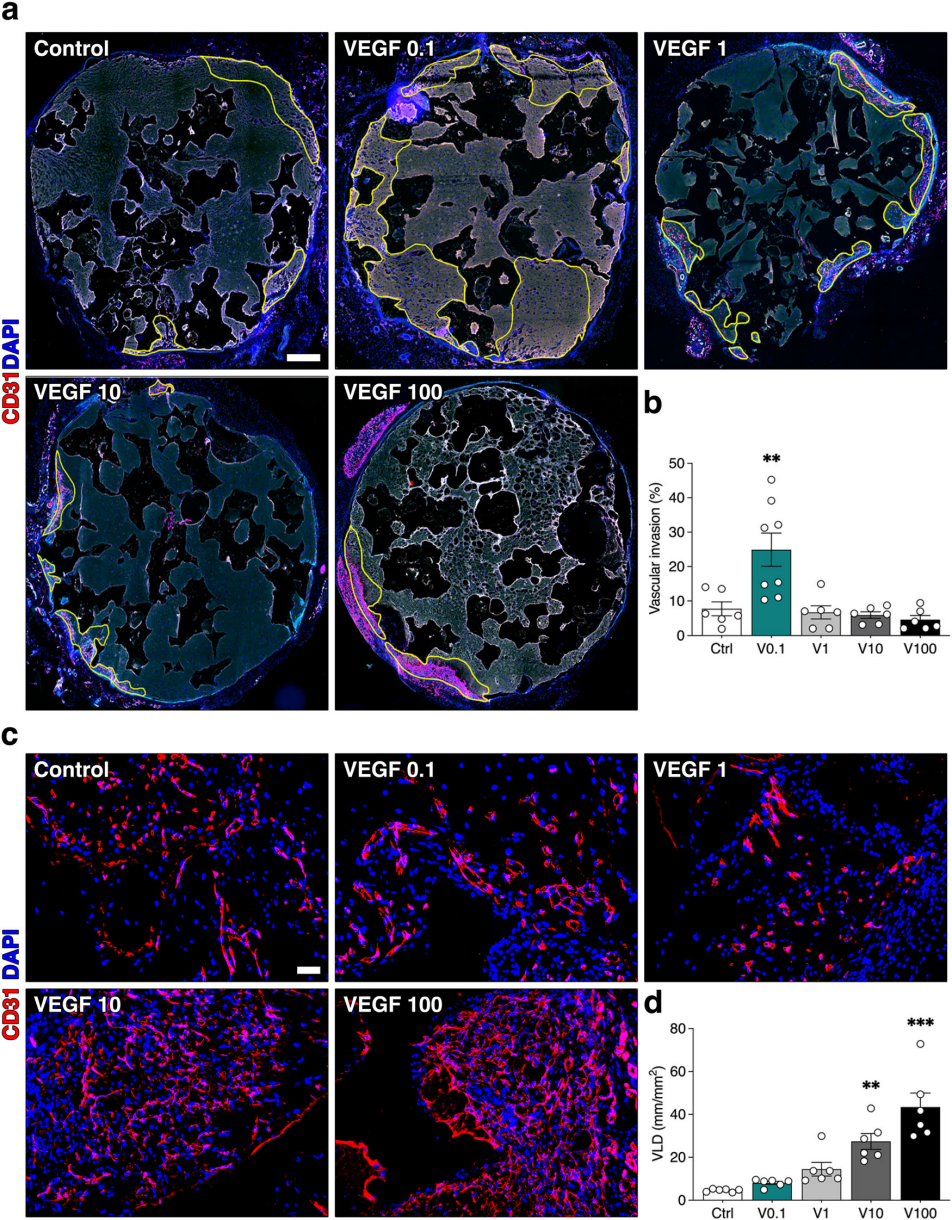
Blood vessel invasion of osteogenic constructs after 1 week of in vivo implantation. **a** Reconstruction of graft sections under fluorescence microscopy to elucidate the areas of blood vessel growth (in yellow), with TG-VEGF doses ranging from 0 to 100 µg/ml; **b** Quantification of the areas invaded by blood vessels (expressed as % of total section area); **c** Assessment of angiogenesis in the invaded areas. Immunostaining of endothelium (CD31, in red) and nuclei (DAPI, in blue) of areas invaded by blood vessels after 1 week of in vivo implantation, with TG-VEGF doses ranging from 0 to 100 µg/ml; **d** Quantification of induced angiogenesis, after 1 week in vivo, expressed as VLD (vessel length density), calculated as millimeters of vessel length per square millimeter of tissue area (mm/mm^2^). Values are expressed as mean ± s.e.m.; **p < 0.01, ***p < 0.001. *N* = 6–8. Scale bars (**a**) = 500 µm; (**c**) = 100 µm.

First, we addressed the effects of VEGF dose on angiogenesis in osteogenic grafts. Invasion by host blood vessels needs to occur rapidly within the first week after in vivo implantation to ensure progenitor survival. Therefore, osteogenic constructs were generated with fibrin decorated with increasing doses of TG-VEGF (0.1, 1, 10 and 100 µg/ml) or without recombinant factor as control, together with 1 × 10^6^ human bone marrow-derived stromal cells (BMSC) per construct (Fig. 1). To control the rate of fibrin degradation and ensure it would take longer than 4 weeks, a TG-version of the fibrinolysis inhibitor aprotinin was included at a concentration of 51 µg/ml, as previously determined^23^. After 1 week, in vivo vascular ingrowth was assessed by quantifying areas invaded by host blood vessels on whole-section microscopy reconstructions (yellow tracings of CD31 + areas in Fig. 2a). As expected, vascular growth started from the surrounding tissue and remained confined at the periphery of the control constructs, while the presence of 0.1 µg/ml of TG-VEGF significantly increased invasion up to about 25% of the construct area (Fig. 2b). However, higher doses of TG-VEGF not only did not further increase invasion, but actually prevented it, as vascularized areas were similar in size to the control condition with no VEGF (Fig. 2b; TG-VEGF 0.1 = 24.9 ± 4.8% vs Control = 7.7 ± 4.9%, *p* < 0.01; and TG-VEGF 1 = 6.7 ± 1.9%, TG-VEGF 10 = 5.9 ± 0.9%, TG-VEGF 100 = 4.5 ± 1.2%; *p* = n.s. vs Control).

In order to determine whether increasing TG-VEGF doses actually impaired vascular growth or rather vessel migration into the constructs, vascular density was assessed within the invaded areas by quantification of vessel length density (VLD), obtained by tracing individual vascular structures and defined as millimeters of vessel length per square millimeter of tissue area (Fig. 2c, d). Interestingly, 0.1 µg/ml of TG-VEGF did not increase VLD compared to controls, whereas higher doses progressively increased vascular density within the invaded areas (Control = 4.6 ± 0.3 mm/mm^2^, TG-VEGF 0.1 = 8.1 ± 0.7 mm/mm^2^, TG-VEGF 1 = 14.5 ± 3.2 mm/mm^2^, TG-VEGF 10 = 27.4 ± 3.7 mm/mm^2^, TG-VEGF 100 = 43.4 ± 6.6 mm/mm^2^). Therefore, these results suggest that VEGF dose determines the distribution of induced vessels within the construct, with a low dose favoring rapid ingrowth while maintaining density similar to controls, and higher doses slowing effective ingrowth, thereby increasing vessel density in the periphery.

### Optimal vascular invasion by 0.1 µg/ml TG-VEGF promotes human progenitor proliferation and survival throughout the grafts

**Fig. 3:**
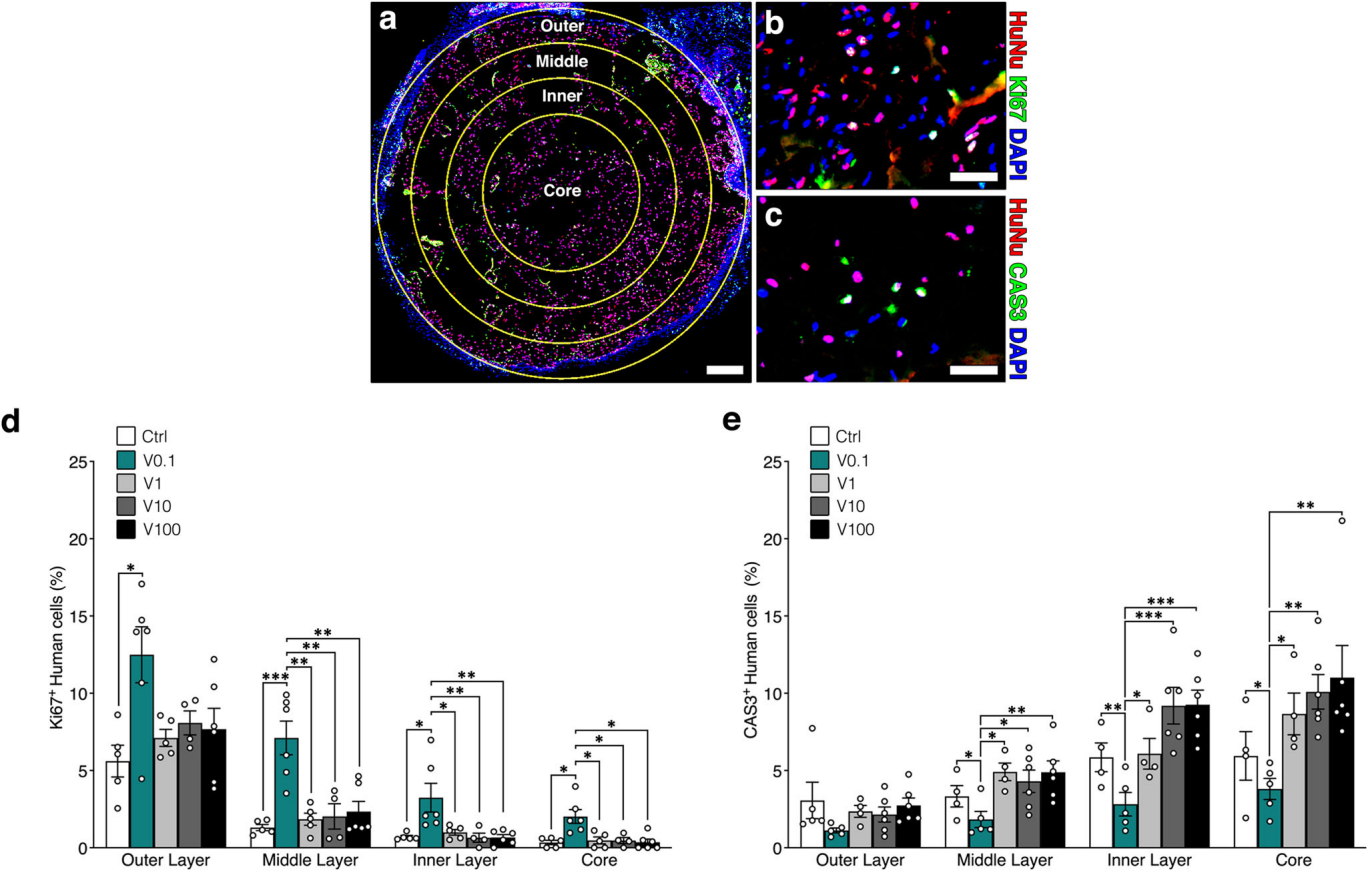
Human BMSC survival and proliferation. **a** Reconstruction of graft sections under fluorescence microscopy (layer subdivision in yellow); **b** Example of immunostaining of human nuclei (HuNu, in red), proliferating cells (Ki67, also in the nucleus, in green) and all nuclei (DAPI, in blue) of constructs (representative picture of the outer layer of TG-VEGF 0.1 μg/ml condition) after 1 week of in vivo implantation; **c** Immunostaining of human nuclei (HuNu, in red), dying cells (CAS3, also in the nucleus, in green) and all nuclei (DAPI, in blue) of constructs (representative picture of the middle layer of TG-VEGF 10 μg/ml condition) after 1 week of in vivo implantation **d** Quantification of proliferating human cells (%) in each layer. **e** Quantification of dying human cells (%) in each layer. Values are expressed as mean ± s.e.m.; *p < 0.05, **p < 0.01, ***p < 0.001. *N* = 4–6. Scale bars (**a**) = 500 µm; (**b**, **c**) = 50 µm.

To investigate the functional effects of the rapid vascular invasion provided by a low dose (0.1 μg/ml) of TG-VEGF, proliferation and apoptosis of the implanted human cells were systematically analyzed 1 week after implantation at different levels of depth inside the graft, by drawing 3 concentric layers, each spanning a depth of 500 µm, and a central core covering the last 1 mm till the center (Fig. 3a). Human cells were identified by staining for a specific human nuclear protein (HuNu), while proliferating and apoptotic cells were recognized by Ki67 or Cleaved-Caspase3 (Cas3) staining, respectively (Fig. 3b, c). A clear trend of decreasing proliferation was observed at increasing depths towards the centre of the graft in each condition, with the notable exception of the 0.1 µg/ml dose of TG-VEGF, which significantly increased the proportion of proliferating human progenitors in each layer compared to all other conditions (Fig. 3d). Interestingly, even deep inside the core, grafts containing 0.1 µg/ml of TG-VEGF enabled human progenitors to proliferate similarly to the middle layer of all other conditions (TG-VEGF 0.1 = 2.0 ± 0.4% Ki67^+^ human cells vs 1–2% for all other conditions in the middle layer), despite being about 1 mm deeper (Core depth = 1.5–2.5 mm; Middle layer depth = 0.5–1 mm).

Conversely, quantification of Cas3^+^ human cells showed a clear trend of increasing apoptosis, for each condition, from the surface towards the core of the graft (Fig. 3e). While no significant difference between the conditions was observed in the outer layer of the grafts, only 0.1 μg/ml of TG-VEGF significantly promoted human cells survival in all deeper layers down to the core, never exceeding the frequency of apoptotic cells of the outer layer (TG-VEGF 0.1 Core = 3.8 ± 0.7% Cas3^+^ human cells vs 2–3% for all other conditions in the outer layer).

### Steady-state vascular density increases directly with VEGF dose

**Fig. 4:**
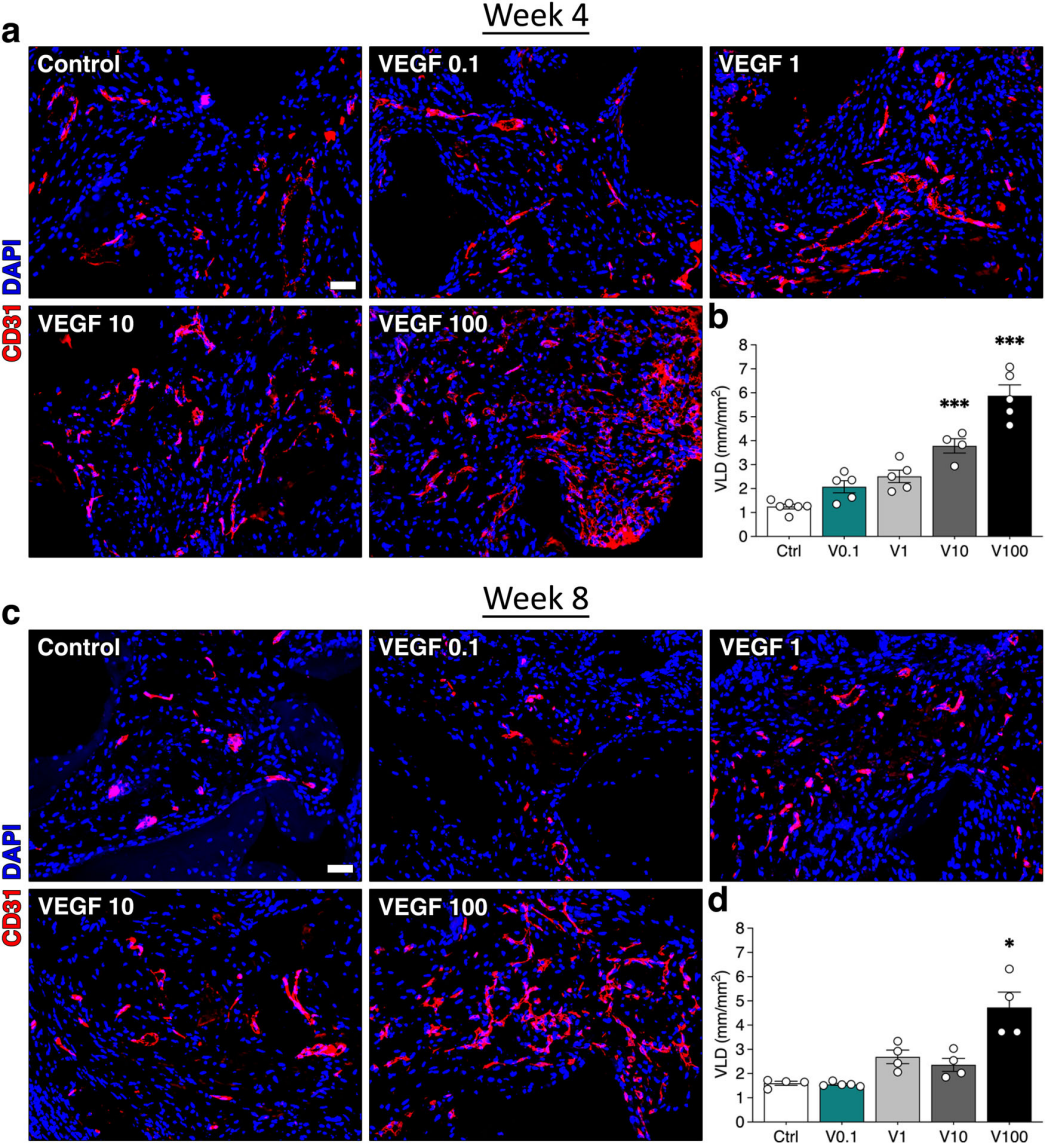
Long term vascularization of osteogenic constructs. **a**, **c** Immunostaining of endothelium (CD31, in red) and nuclei (DAPI, in blue) of constructs, with TG-VEGF doses ranging from 0 to 100 µg/ml, after 4 (**a**) and 8 (**c**) weeks of in vivo implantation. **b**, **d** Quantification of vessel length density (VLD), expressed as millimeters of vessel length per square millimeter of tissue area (mm/mm^2^) after 4 (**b**) and 8 (**d**) weeks of in vivo implantation. Values are expressed as mean ± s.e.m.; *p < 0.05, ***p < 0.001. *N* = 4–6. Scale bars = 100 μm.

Four weeks after in vivo implantation, constructs were completely invaded by blood vessels in all conditions, as expected. At this late time-point quantification of VLD (Fig. 4a, b) showed that global vascular density was progressively increased by increasing TG-VEGF doses starting from 1 μg/ml compared to controls (Control = 1.3 ± 0.1 mm/mm^2^, TG-VEGF 0.1 = 2.1 ± 0.3 mm/mm^2^ *p* = n.s., TG-VEGF 1 = 2.5 ± 0.3 mm/mm^2^ *p* < 0.05; TG-VEGF 10 = 3.8 ± 0.3 mm/mm^2^ *p* < 0.001; TG-VEGF 100 = 5.9 ± 0.5 mm/mm^2^ *p* < 0.001). After 8 weeks, vascular density remained similar to what was observed at the 4 week time-point (Fig. 4c, d; Control = 1.6 ± 0.1 mm/mm^2^, TG-VEGF 0.1 = 1.6 ± 0.1 mm/mm^2^, TG-VEGF 1 = 2.7 ± 0.3 mm/mm^2^; TG-VEGF 10 = 2.4 ± 0.3 mm/mm^2^; TG-VEGF 100 = 4.7 ± 0.6 mm/mm^2^ *p* < 0.001). These results show that the steady-state had been reached by 4 weeks and that the induced vasculature was stable.

### Increasing VEGF dose progressively impairs bone tissue formation

**Fig. 5:**
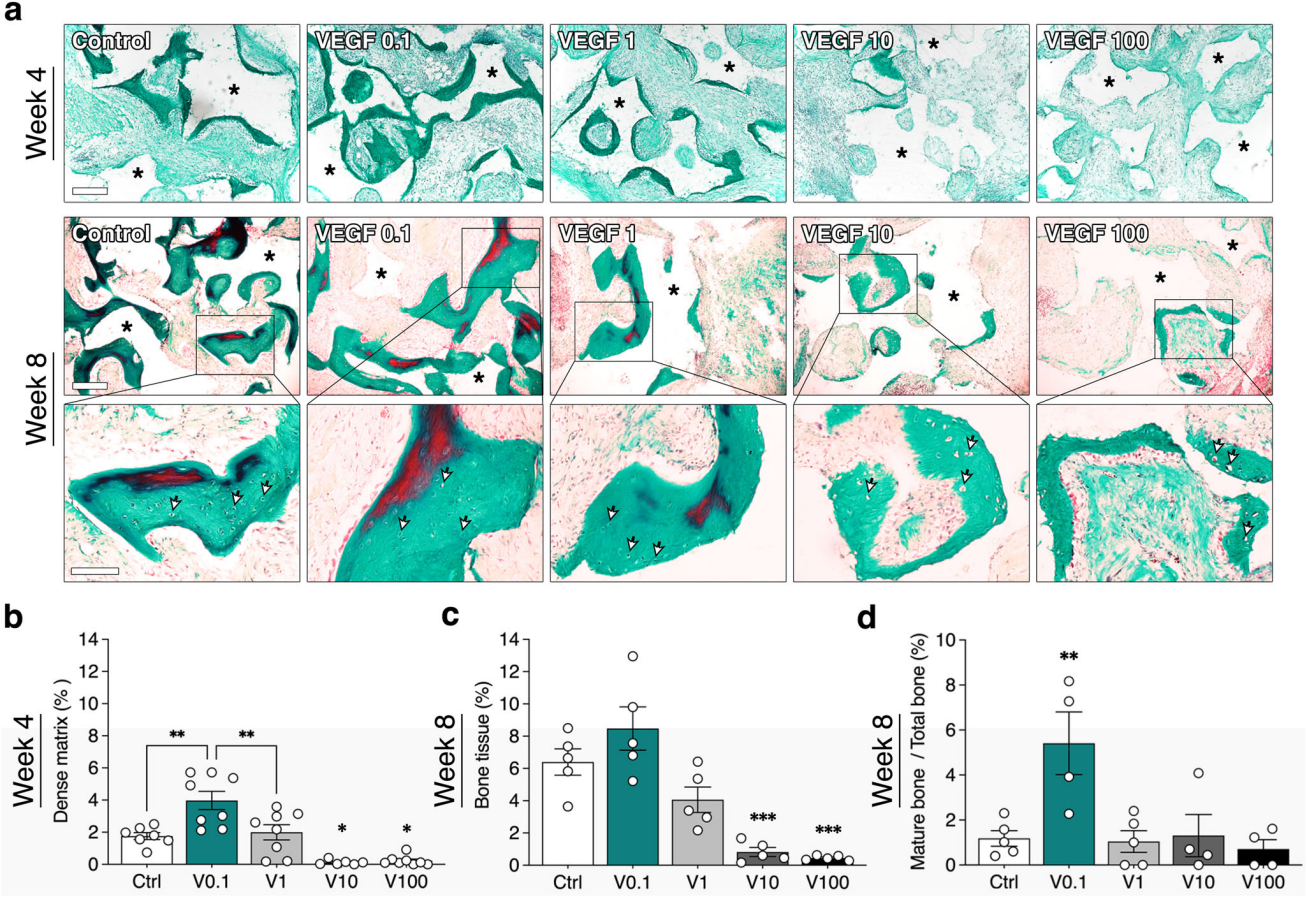
Bone tissue formation and maturation. **a** Representative images of Masson’s trichrome staining of constructs 4 weeks (top panels) and 8 weeks (bottom panels) after in vivo implantation (dense collagenous tissue in green, elastic fibers in red), with TG-VEGF doses ranging from 0 to 100 µg/ml (Scale bars = 200 µm). Higher magnification images are shown in the bottom row (Scale bars = 100 µm). Asterisks = hydroxyapatite granules; arrowheads = osteocyte lacunae. **b** Quantification of areas occupied by dense collagenous matrix at 4 weeks (expressed as % of construct area). **c** Quantification of areas occupied by bone tissue at 8 weeks (expressed as % of construct area). **d** Quantification of areas occupied by mature bone at 8 weeks (expressed as % of total bone tissue). Values are expressed as mean ± s.e.m.; *p < 0.05, **p < 0.01, ***p < 0.001. *N* = 4–6.

Next, we investigated the effects of VEGF dose on bone formation. Bone matrix deposition was assessed with Masson’s trichrome staining, which shows the presence of compact collagen fibers as well as of elastic fibers characteristic of mature bone. After 4 weeks in vivo, fibrin was almost completely degraded, but still present, and initial formation of a dense collagenous matrix could be observed at the interface with the hydroxyapatite granules (dark green stain in Fig. 5a top panels). However, grafts with TG-VEGF doses higher than 1 µg/ml contained almost only fibrous tissue. Quantification of the areas occupied by dense collagenous matrix (Fig. 5b) showed that 0.1 µg/ml TG-VEGF significantly promoted matrix deposition compared to controls (Control = 1.8 ± 0.2%, TG-VEGF 0.1 = 4.0 ± 0.6%; *p* < 0.01). However, 1 µg/ml TG-VEGF negated this improvement (TG-VEGF 1 = 2.0 ± 0.5%; *p* = n.s. vs Control and *p* < 0.01 vs TG-VEGF 0.1) and higher TG-VEGF doses almost completely prevented dense matrix deposition (TG-VEGF 10 = 0.1 ± 0.1%, TG-VEGF 100 = 0.2 ± 0.1%; *p* < 0.05 vs Control).

After 8 weeks, frank bone tissue could be observed, characterized by dense collagenous matrix with organized collagen fibers and the presence of osteocyte lacunae (Fig. 5a, bottom panels; arrowheads indicate some lacunae). Bone formation was severely and dose-dependently impaired by TG-VEGF doses higher that 1 µg/ml (Fig. 5c: Control = 6.4 ± 0.8%, TG-VEGF 10 = 0.8 ± 0.3%, TG-VEGF 100 = 0.5 ± 0.1%; *p* < 0.001 vs Control), whereas the 0.1 and 1 µg/ml doses supported bone tissue formation similarly to the controls (TG-VEGF 0.1 = 8.5 ± 1.3%, TG-VEGF 1 = 4.1 ± 0.8%; *p* = n.s. vs Control). Interestingly, although the differences were not statistically significant due to intrinsic variability, a clear trend could be observed also at 8 weeks for 0.1 µg/ml to enable better bone formation than controls, while 1 µg/ml caused already an initial loss.

The degree of maturation of bone tissue can be evaluated by the presence of elastic fibers, shown in red by Masson’s trichrome staining. Quantification of the areas occupied by elastic fibers (Fig. 5d, expressed as percentage of total bone tissue formed) showed that that the 0.1 µg/ml TG-VEGF dose could significantly increase the amount of mature bone tissue compared to all other conditions (TG-VEGF 0.1 = 5.4 ± 1.4% vs Control = 1.2 ± 0.3%, TG-VEGF 1 = 1.1 ± 0.5%, TG-VEGF 10 = 1.3 ± 0.9%, TG-VEGF 100 = 0.7 ± 0.4%; *p* < 0.05).

### Increasing VEGF dose stimulates osteoclastic bone resorption

**Fig. 6:**
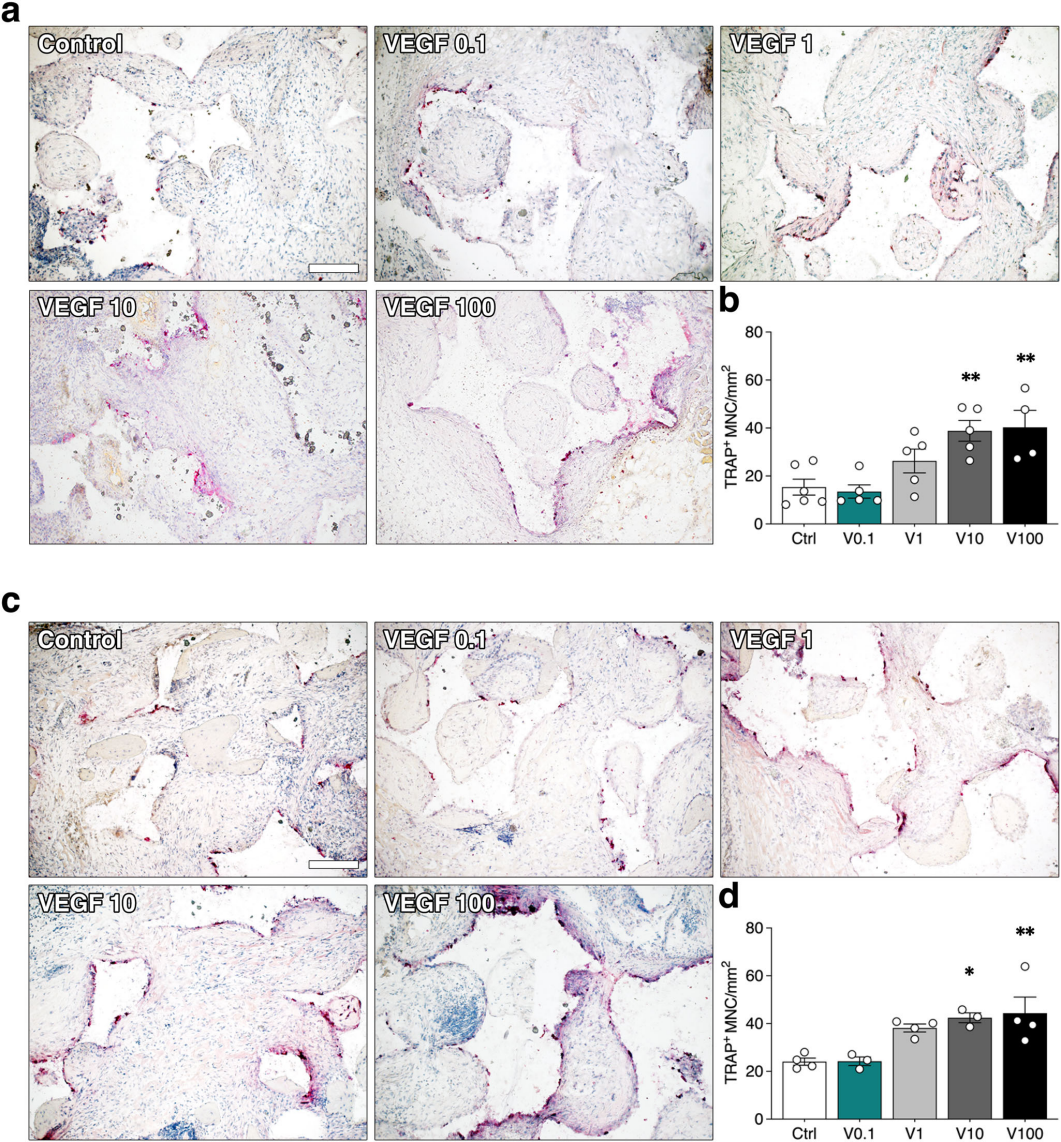
Osteoclast recruitment. **a***,* **c** Histochemical stain for TRAP activity (red) and nuclear counterstaining with hematoxylin (blue) of constructs 4 weeks (**a**) and 8 weeks (**c**) after in vivo implantation, with TG-VEGF doses ranging from 0 to 100 µg/ml; **b**, **d** Quantification of Trap^+^ multinucleated cells (MNC) per tissue area (mm^2^) after 4 (**c**) and 8 (**d**) weeks. Values are expressed as mean ± s.e.m.; *p < 0.05, **p < 0.01. *N* = 3–6. Scale bar = 200 µm.

The amount of bone tissue depends on the balance between deposition by osteoblasts and resorption by osteoclasts. Therefore, osteoclast recruitment was assessed by staining for the osteoclast-specific enzyme tartrate-resistant acid phosphatase (TRAP). Few TRAP + cells were detected in the constructs with BMSC alone or in combination with 0.1 µg/ml TG-VEGF, in close proximity to the bone matrix or at the interface with the hydroxyapatite granules, both at 4 and 8 weeks (Fig. 6a, c). Quantification of the number of TRAP^+^ multinucleated cells per tissue area (TRAP^+^ MNC/mm^2^) showed that TG-VEGF doses higher than 1 µg/ml significantly increased osteoclast recruitment at both time points (Fig. 6b, d; 4 weeks: Control = 15.3 ± 3.3 TRAP^+^ MNC/mm^2^, TG-VEGF 10 = 38.8 ± 4.3, TG-VEGF 100 = 40.3 ± 7.1, *p* < 0.01 vs Control; 8 weeks: Control = 24.1 ± 1.5 TRAP^+^ MNC/mm^2^, TG-VEGF 10 = 42.4 ± 2.1, TG-VEGF 100 = 44.4 ± 6.7, *p* < 0.05 and <0.01 vs Control, respectively). Constructs containing 0.1 µg/ml of TG-VEGF, instead, did not increase osteoclast recruitment compared to controls, whereas 1 µg/ml showed a non-significant trend towards increase (TG-VEGF 0.1 = 13.5 ± 2.8, TG-VEGF 1 = 26.3 ± 4.9 at 4 weeks and TG-VEGF 0.1 = 24.2 ± 1.8 and TG-VEGF 1 = 38.2 ± 1.6 at 8 weeks, *p* = n.s. vs Control).

### A minimum dose of 0.1 µg/ml TG-VEGF is required to couple accelerated vascular invasion and improved bone formation

**Fig. 7:**
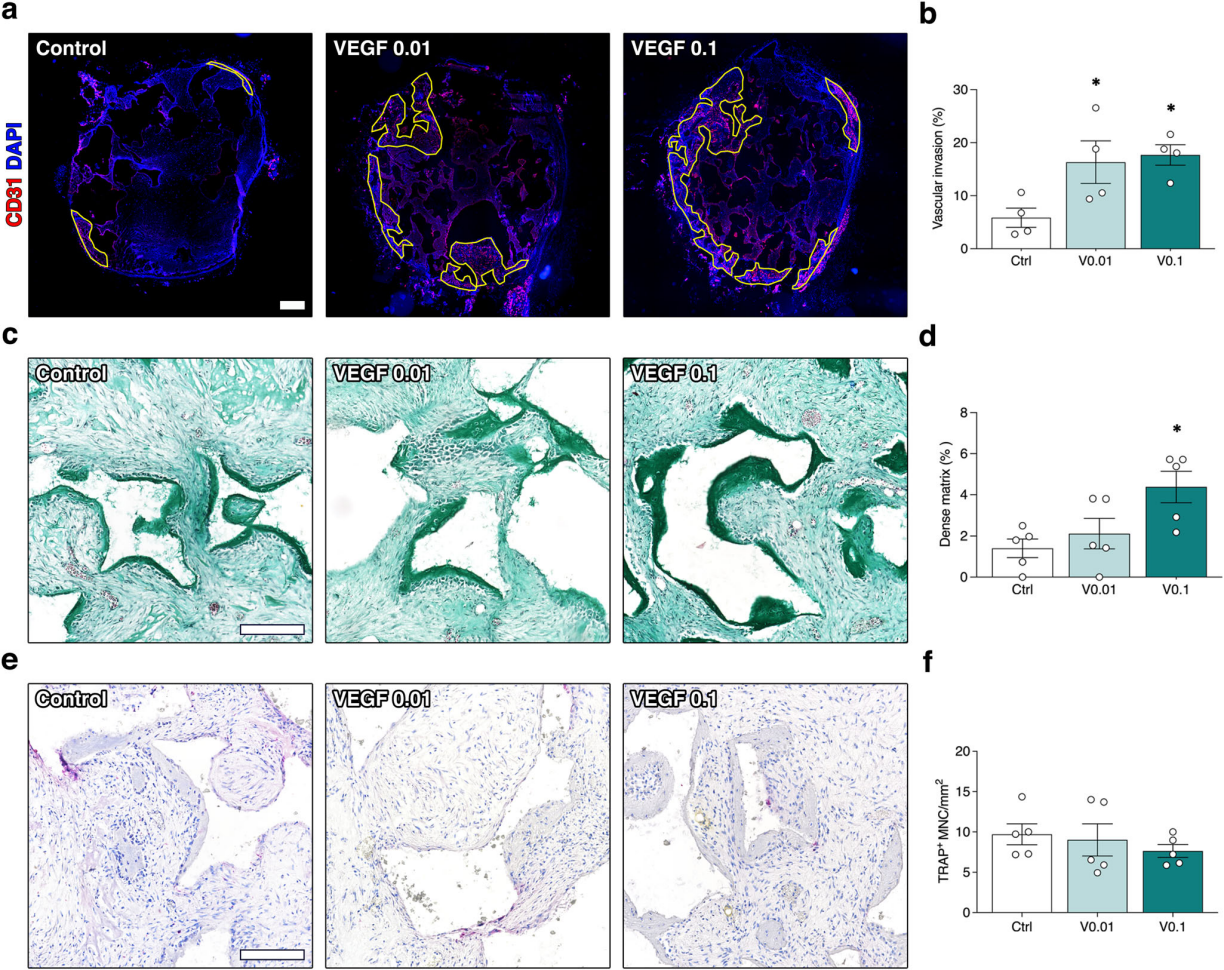
Early vascular invasion, bone formation and osteoclast recruitment by low VEGF doses. **a** Reconstruction of graft sections under fluorescence microscopy to elucidate the areas of blood vessel growth (in yellow), with TG-VEGF doses ranging from 0 to 0.1 µg/ml; **b** Quantification of the areas invaded by blood vessels (expressed as % of total section area); **c** Bone tissue formation. Representative images of Masson’s trichrome staining of constructs 4 weeks after in vivo implantation (dense collagenous tissue in green), with TG-VEGF doses ranging from 0 to 0.1 µg/ml. **d** Quantification of areas occupied by dense collagenous matrix at 4 weeks (expressed as % of construct area). **e** Osteoclast recruitment. Histochemical stain for TRAP activity (red) and nuclear counterstaining with hematoxylin (blue) of constructs 4 weeks after in vivo implantation, with TG-VEGF doses ranging from 0 to 0.1 µg/ml; **f** Quantification of Trap^+^ multinucleated cells (MNC) per tissue area (mm^2^) after 4 weeks. Values are expressed as mean ± s.e.m.; *p < 0.05. *N* = 5. Scale bars = 200 µm.

Based on the observed negative effects of increasing TG-VEGF doses on both vascularization and bone formation, we investigated whether further improvements could be achieved with an even lower dose. Constructs decorated with 0.01 µg/ml of TG-VEGF were implanted and analyzed as above after 1 and 4 weeks in vivo. As shown in Fig. 7a, b, after 1 week 0.01 µg/ml of TG-VEGF significantly increased invasion of the construct area compared to controls and similarly to 0.1 µg/ml (Fig. 7b; Control = 5.9 ± 1.8%, TG-VEGF 0.01 = 16.3 ± 4.0%, TG-VEGF 0.1 = 17.7 ± 1.9%; *p* < 0.05 both vs Control). On the other hand, after 4 weeks the gain in osteogenic matrix deposition observed with 0.1 µg/ml of TG-VEGF was completely lost by lowering the dose to 0.01 µg/ml (Fig. 7c, d; Control = 1.4 ± 0.5%, TG-VEGF 0.01 = 2.1 ± 0.7%, TG-VEGF 0.1 = 4.4 ± 0.8%; *p* = n.s. TG-VEGF 0.01 vs Control). Lastly, osteoclast recruitment was not affected by 0.01 µg/ml of TG-VEGF compared to either controls or 0.1 µg/ml of TG-VEGF (Fig. 7e, f; Control = 9.7 ± 1.3 TRAP^+^ MNC/mm^2^, TG-VEGF 0.01 = 9.0 ± 1.9, TG-VEGF 0.1 = 7.6 ± 0.8; *p* = n.s.). Therefore, these data show that a minimum dose of TG-VEGF (0.1 µg/ml) is required to stimulate bone formation in association with accelerated vascular invasion.

### VEGF dose specifically regulates osteogenic differentiation of human progenitors in vivo and induction of pro-osteogenic endothelium

**Fig. 8:**
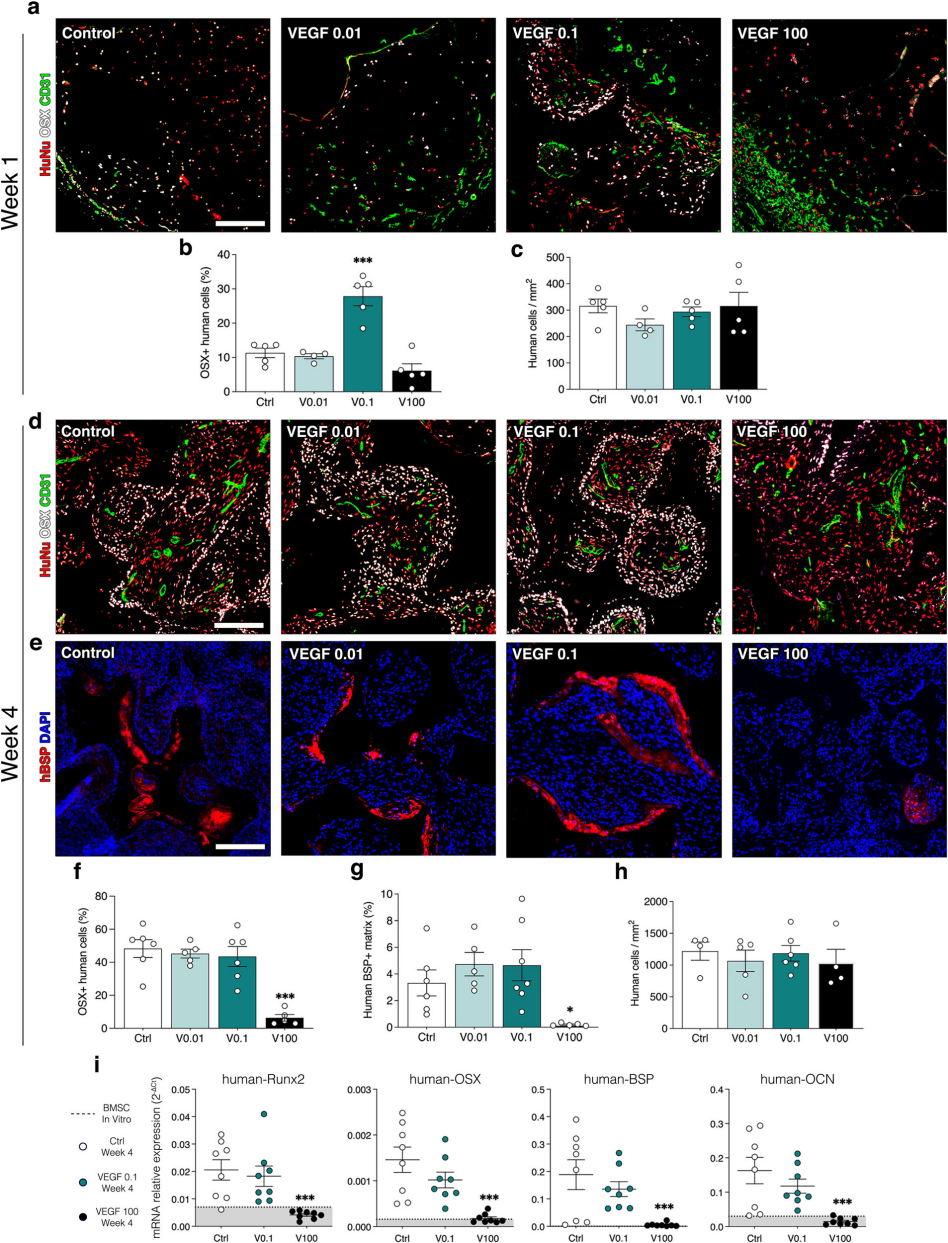
Human osteoprogenitor differentiation in vivo. **a**, **d** Immunostaining of osteogenic constructs 1 week (**a**) and 4 weeks (**d**) after in vivo implantation with TG-VEGF doses ranging from 0 to 100 µg/ml for Osterix (OSX, white), human nuclei (HuNu, red) and blood vessels (CD31, green). **b**, **f** Quantification of the number of Osx positive human cells after 1 week (**b**) and 4 weeks (**f**) expressed as % of the total number of human cells. **c**, **h** Quantification of the number of human cells per tissue area (mm^2^) after 1 week (**c**) and 4 weeks (**h**) (*n* = 5–6); **e** Immunostaining of osteogenic constructs 4 weeks after in vivo implantation for human BSP (hBSP, red) and nuclei (DAPI, blue). **g** Quantification of human BSP positive matrix after 4 weeks expressed as percentage of total tissue area (*n* = 5–6). (**i**) Gene expression of human Runx2, OSX, BSP and OCN was quantified by qRT-PCR and expressed as relative expression to human GAPDH (2^−ΔCt^), with TG-VEGF doses ranging from 0 to 100 µg/ml. Data represent the values of individual samples and the mean (black bar, *n* = 8); dotted line and grey area = expression range of undifferentiated human BMSC in vitro. Values are expressed as mean ± s.e.m.; *p < 0.05, ***p < 0.001. Scale bars = 200 µm.

We also investigated the influence of VEGF dose on in vivo osteogenic differentiation of human BMSC. Since bone differentiation is a progressive process, requiring progenitor commitment and osteoblast expansion before the deposition of bony matrix in vivo, BMSC commitment was assessed by measuring expression of the early osteogenic transcription factor Osterix (Osx) in human cells after 1 week in vivo. Based on the results of bone formation, constructs decorated with 0.01, 0.1 and 100 µg/ml of TG-VEGF were selected for the comparison. As shown in Fig. 8a, a significant proportion of human progenitors already underwent osteogenic commitment after 1 week and they were specifically localized in a few concentric layers around the invading vascular front. In agreement with the bone formation data above, 0.1 µg/ml significantly increased the amount of Osx^+^ human progenitors, whereas 0.01 µg/ml did not provide any improvement compared to controls (Fig. 8b). On the other hand, a high dose of 100 µg/ml already showed a trend towards loss of osteogenic commitment (Control = 11.3 ± 1.4%, TG-VEGF 0.01 = 10.3 ± 0.7%, TG-VEGF 0.1 = 27.9 ± 2.8%, TG-VEGF 100 = 6.1 ± 2.0%; *p* < 0.001 TG-VEGF 0.1 vs Control). We sought to determine whether VEGF specifically impaired the osteogenic differentiation of human BMSC or their survival. The number of human cells was quantified and it was found to remain similar among all conditions (Fig. 8c; Control = 316.2 ± 26.0 cells/mm^2^, TG-VEGF 0.01 = 244.4 ± 22.4, TG-VEGF 0.1 = 293.9 ± 18.5 cells/mm^2^, TG-VEGF 100 = 315.5 ± 52.1 cells/mm^2^; *p* = n.s.).

In order to investigate whether high VEGF might simply delay osteogenic commitment rather than blocking it, we measured the expression of both Osx and the later osteogenic marker Bone Sialoprotein (BSP) in constructs decorated with 100 µg/ml of TG-VEGF 4 weeks after implantation and compared it to the osteogenic conditions 0.1 and 0.01 µg/ml and controls. Immunostaining showed that 100 µg/ml of TG-VEGF caused a significant loss of both osteogenic proteins, whereas their expression was preserved with both 0.01 and 0.1 µg/ml (Fig. 8d–g; hOsx^+^ cells: Control = 48.3 ± 5.4%, TG-VEGF 0.01 = 45.3 ± 2.7%, TG-VEGF 0.1 = 43.5 ± 6.1%, TG-VEGF 100 = 6.4 ± 2.0% of human cells, *p* < 0.001; hBSP^+^ matrix: Control = 3.3 ± 0.9%, TG-VEGF 0.01 = 4.7 ± 0.9%, TG-VEGF 0.1 = 4.7 ± 1.2%, TG-VEGF 100 = 0.2 ± 0.1%, *p* < 0.05). Also at this later time-point the number of human cells was found to remain similar among all conditions (Fig. 8h; Control = 1220 ± 143.6 cells/mm^2^, TG-VEGF 0.01 = 1066 ± 169.1 cells/mm^2^, TG-VEGF 0.1 = 1186 ± 123.7 cells/mm^2^, TG-VEGF 100 = 1036 ± 212.5 cells/mm^2^; p = n.s.). Lastly, we expanded these data by in vivo gene expression analysis of 2 early osteogenic transcription factors (Runx2 and Osx) and 2 later bone matrix proteins (BSP and Osteocalcin, OCN). As shown in Fig. 8i, all osteogenic genes were strongly upregulated in constructs containg BMSC alone as well as with 0.1 µg/ml of TG-VEGF, indicating robust differentiation. However, 100 µg/ml of TG-VEGF significantly impaired the upregulation of all osteogenic gene expression, which was barely greater than the undifferentiated controls.

Therefore, a high VEGF dose did not affect initial osteoprogenitor survival and engraftment, but specifically interfered with their osteogenic commitment, providing a further mechanism for the observed impairment of bone tissue formation by high VEGF doses. On the other hand, a minimum amount of VEGF (0.1 µg/ml) was required to specifically promote osteogenic commitment of human progenitors.

**Fig. 9:**
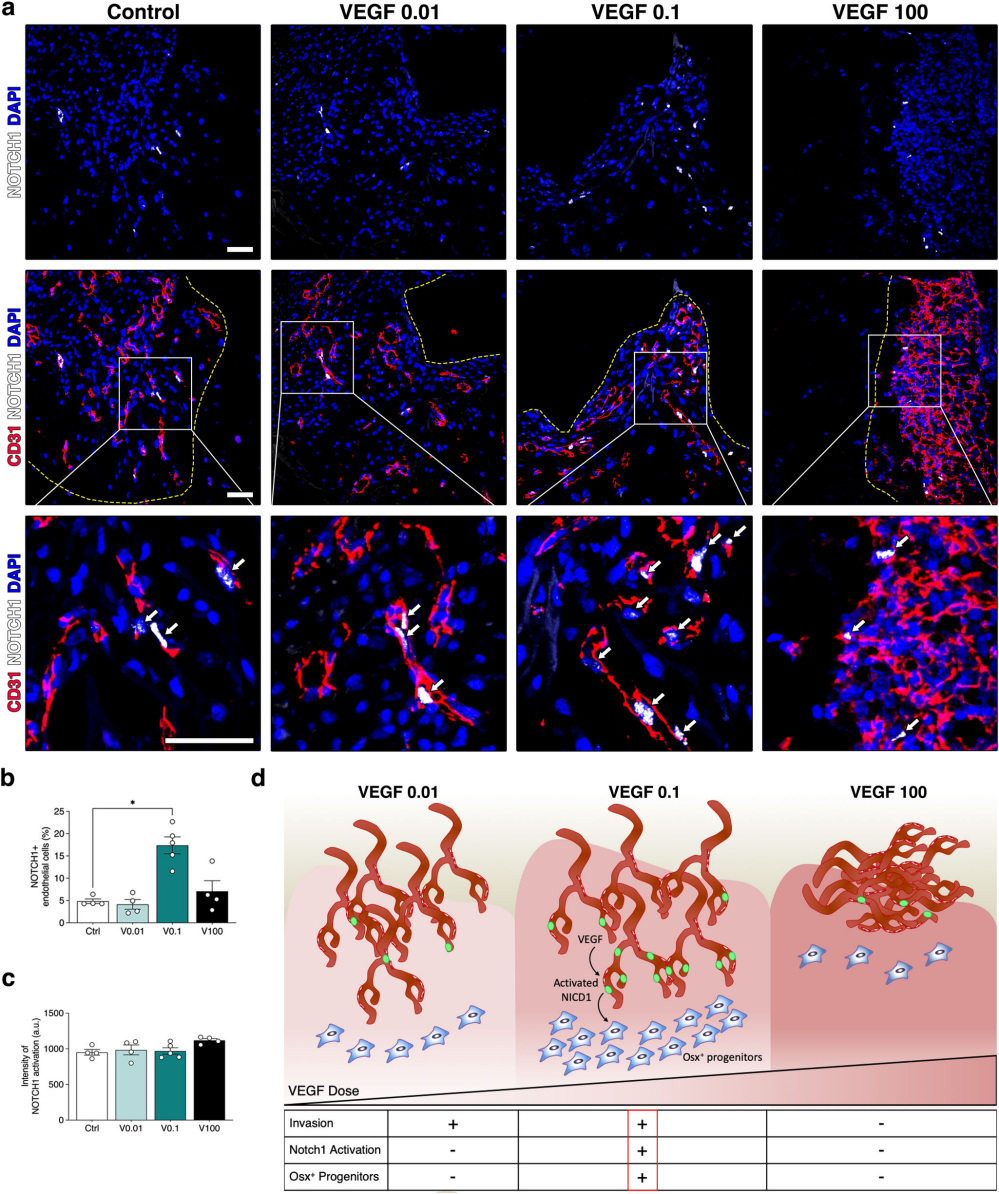
Induction of Notch1 + pro-osteogenic endothelium. **a** Immunostaining of osteogenic constructs 1 week after in vivo implantation with TG-VEGF doses ranging from 0 to 100 µg/ml, showing activated Notch1 (intranuclear white signal), blood vessels (CD31, red) and nuclei (DAPI, blue). The yellow dashed lines indicate the limit of the vascular invasion fronts. The bottom panels show high-magnification images of the areas indicated in the middle panels and white arrows indicate endothelial nuclei with activated Notch1. **b** Quantification of the number of endothelial nuclei with activated Notch1, expressed as % of the total number of endothelial cells. **c** Quantification of the mean intensity of intranuclear activated Notch1 signaling (in arbitrary units, a. u.). **d** Cartoon summarizing the regulation of vascular invasion, Notch1 activation (green nuclei) and osteogenic commitment (Osx^+^) of progenitors by VEGF dose. In the underlying table, - means no improvement and + means significant improvement compared to control. Values are expressed as mean ± s.e.m.; *p < 0.05. Scale bars = 50 µm.

As shown in Fig. 8a, Osx^+^ cells could only be found in the vicinity of the invading vascular front. Interestingly, both 0.01 and 0.1 µg/ml of VEGF caused similar vascular invasion, but only 0.1 µg/ml improved osteogenic differentiation. This suggests a functional difference in the endothelium stimulated by the two doses. The pro-osteogenic function of blood vessels has been shown to depend on the production of angiocrine signals by a specialized endothelial phenotype named Type H^24^, which is mechanistically induced by activation of Notch1 signaling^25^. Therefore, we investigated whether VEGF dose regulated endothelial Notch1 activation in osteogenic constructs. For this, we used an antibody specifically recognizing the cleaved form of Notch1 intracellular domain (NICD1), which upon activation is translocated into the nucleus where it initiates its specific transcriptional response, together with a co-staining for CD31 to positively identify endothelial cells of invading vascular structures. As shown in Fig. 9a, after 1 week the ingrowing endothelium at the invasion front in both controls and constructs with 0.01 µg/ml of TG-VEGF showed very rare Notch1^+^ nuclei, whereas 0.1 µg/ml increased Notch1 activation by almost 4-fold. Instead, this increase was lost with a high dose of 100 µg/ml (Fig. 9b; Notch1^+^ endothelial cells: Control = 4.8 ± 0.5%, TG-VEGF 0.01 = 4.1 ± 1.1%, TG-VEGF 0.1 = 17.4 ± 1.9%, TG-VEGF 100 = 7.1 ± 2.4%; *p* < 0.05 TG-VEGF 0.01 vs control). We further assessed the degree of Notch1 activation in the positive endothelial nuclei by quantification of the mean signal intensity and no significant differences among conditions were observed (Fig. 9c).

## Discussion

Taking advantage of a unique and highly tunable platform, here we could dissect how VEGF dose over a 1’000-fold range controls the intimately connected processes of angiogenesis and osteogenesis in the generation of engineered bone. We found that increasing VEGF dose not only impairs bone formation, but surprisingly also delays vascular invasion of the engineered constructs. However, stimulation of early osteogenic commitment and bone formation required a minimum VEGF dose in the graft microenvironment (0.1 µg/ml), which promoted the specific induction of a pro-osteogenic endothelial phenotype, marked by activation of Notch1 signaling. Therefore, the effective coupling of optimal osteogenesis and rapid vascular invasion is finely regulated by VEGF dose (Fig. 9d). The underlying mechanism is complex and comprises distinct and opposing effects on vessel migration and endothelial function, bone resorption through osteoclast recruitment and bone formation through osteoprogenitor differentiation.

An explanation for the unexpected effects of VEGF dose on vascular invasion lies in the dynamic nature of construct vascularization. In fact, upon in vivo implantation, blood vessel in-growth must be stimulated initially from the surrounding pre-existing vascular network, while later the vascular density in the invaded areas is regulated by the metabolic needs of the tissue. Angiogenesis is commonly quantified as steady-state vascular density within the implant, without taking in consideration the kinetics of growth. What we found here is that, although the final vascular density is indeed positively regulated by VEGF dose, the speed of vascular invasion is instead impaired by increasing VEGF dose. This is a very important distinction. In fact, we found that rapid vascularization is the key parameter to improve progenitor survival and proliferation, as shown in Fig. 3. On the other hand, doses that increased vascular density, but not early invasion, failed to do so, showing that stimulating vessel density beyond its physiological values is not required to support the metabolic needs of this developing tissue. Therefore, for the therapeutic purpose of promoting net bone formation, it is important to target rapid vascular ingrowth with a low VEGF dose rather than increasing vessel density with a higher one.

The observation that higher VEGF dose impaired active vascular migration inside the graft and rather promoted vessel expansion at the graft surface, leading to high vascular densities in limited areas, is intriguing. A consideration of the different mechanisms by which new vessels may grow suggests an explanation for this phenomenon. In fact, rapid invasion of an avascular tissue, such as an implanted graft, takes place by the process of vascular sprouting^26^. Sprouting angiogenesis is guided by the formation of VEGF concentration gradients and requires coordinated endothelial migration and proliferation to enter the tissue^27,28^. Even though the constructs were decorated with homogeneous concentrations of VEGF, it is interesting to note that micro-gradients do actually form with low VEGF doses upon fibrin degradation. In fact, VEGF_164_ has an intermediate degree of affinity for ECM and, when released from fibrin, is able to undergo limited diffusion^29^. However, vascular growth can also take place by the alternative mechanism of intussusception, or splitting angiogenesis. In this case, endothelial cells proliferate without migrating, leading to circumferential enlargement of pre-existing vessels, which subsequently split longitudinally to form new structures^30–32^. This mode of vascular growth is highly efficient to expand pre-existing networks, but does not have an intrinsic directional component, leading to increases in vessel density rather than to invasion. Recent unpublished findings by our group suggest that splitting angiogenesis results from higher doses of VEGF, which saturate the extracellular matrix of the vessel microenvironment and therefore present endothelial cells with a flat concentration profile rather than a gradient (Gianni-Barrera, R.; Banfi, A. et al. manuscript in preparation). This could explain the observed delay in vascular invasion of grafts decorated with concentrations greater than 1 µg/ml of TG-VEGF.

The initial discrepancy in vascular invasion was a transient phenomenon, as all grafts were fully vascularized with all VEGF doses after 4 weeks. A likely explanation is related to the transient nature of the fibrin-bound delivery of VEGF: as fibrin degrades over this time-frame, the “barrier” of high VEGF concentration also is lost and the endothelium is again exposed to moderate VEGF concentrations and microenvironmental gradients conducive to migration inside the tissue. Therefore, high VEGF doses do not stably prevent vascular invasion, but rather delay it over the crucial initial time window of 1 week, nevertheless leading to detrimental effects on progenitor survival/proliferation and bone formation. A similar consideration likely underpins the observation that VEGF-dependent stimulation of osteogenic differentiation of embedded human progenitors is also transient (Fig. 8), consistently with the progressive release of the fibrin-bound VEGF in the localized microenvironments. It should be noted that, although transient, this boost translated into more effective long-term bone formation at both 4 and 8 weeks (Fig. 5).

The effects of different VEGF doses on net bone formation were particularly interesting. In fact, while a dose of 0.1 µg/ml significantly promoted initial bone matrix deposition by 4 weeks compared to controls, both lower and higher doses failed to do so. This observation is strikingly reminiscent of the roles of VEGF during bone development and repair, where its loss causes skeletal deficits and malformations^33,34^, but excessive levels also lead to bone failure^35^. Hu and Olsen^35^ also reported that excessive VEGF delivery can impair endogenous bone repair. In fact, it was found that a concentration of 1’000 µg/ml of VEGF loaded in collagen sponges inhibited intramembranous bone formation in a tibial cortical defect. This was ascribed mainly to reduced collagen I accumulation and BSP expression in the area of the defect, suggesting a possible impairment of endogenous osteoprogenitors differentiation. This non-linear relationship between VEGF dose in the microenvironment and net bone formation likely reflects the complex biological roles of VEGF in both bone anabolism and catabolism. Both osteoprogenitors and osterix-positive osteoblasts express VEGF receptors (especially VEGF-R2) and VEGF can directly regulate their differentiation and activity maintaining bone homeostasis, through paracrine and intracrine mechanisms^36–38^. Here we found that the harmonious association of vascular growth and bone formation is exquisitely dependent on VEGF dose and that different processes are stimulated by distinct dose ranges. In fact, vascular invasion is already stimulated with a very low dose of VEGF (0.01 µg/ml), but promotion of progenitor differentiation requires a higher dose (0.1 µg/ml) and osteoclast recruitment only increases with still higher doses (greater than 1 µg/ml), which also impair vascular invasion (Fig. 9d).

The mechanism by which VEGF regulates both vascular invasion and osteogenic commitment of BMSC is unclear and likely complex. However, a few observations reported here allow some speculation on the nature of this relationship. First, the data reported here suggest that the beneficial effect of angiogenesis on osteogenesis does not depend simply on the physiological function of blood vessels in supplying oxygen and nutrients and removing waste products. In fact, both 0.01 and 0.1 µg/ml of TG-VEGF promoted a similarly effective early vascular invasion of grafts, but osteogenic progenitor commitment could be stimulated only by 0.1 µg/ml. On the other hand, we found evidence against a direct effect of VEGF on the differentiation of implanted BMSC, since progenitors are exposed to the same dose of TG-VEGF throughout the constructs, whereas Osx expression only appears in the vicinity of the vascular front (Fig. 8). Furthermore, Osx induction does not depend on vessel quantity, as higher TG-VEGF doses progressively increase vascular density, but this is actually accompanied by a significant impairment of osteogenic commitment. Therefore, taken together these observations suggest that vessels induced by different doses of VEGF are qualitatively different from each other, beyond the effects on their quantity and kinetics of ingrowth.

These features are highly suggestive of an angiocrine function for the blood vessels induced by different VEGF doses. A specialized Type H endothelial phenotype has been described to regulate bone formation during development and repair^24,25^. Mechanistically, the pro-osteogenic function of Type H endothelium depends on activation of Notch1 signaling that induces the secretion of angiocrine factors such as Noggin^5,25^. Consistently, we found that the pro-osteogenic dose of VEGF (0.1 µg/ml) specifically stimulates Notch1 activation in the endothelium of the invading vascular front (Fig. 9), and the Osx+ osteogenic cells also specifically localize around the invading vessels (Fig. 8). Taken together, these data suggest that an optimal VEGF dose promotes coupled angiogenesis and osteogenesis through the induction of “Type H-like” vessels. However, positive identification will require the discovery of unequivocal Type H markers, which are currently lacking.

In conclusion, here we uncovered how VEGF signaling controls several processes at the crossroads of angiogenesis and osteogenesis in a dose-dependent manner. In a therapeutic perspective, the factor-decorated matrix platform described here provides the ability to precisely control the signaling microenvironment and the translation of these biological findings will need to be tested in an orthotopic critical-size defect model. The molecular players mediating the angiocrine cross-talk between blood vessels and bone stimulated by optimal doses of VEGF remains to be elucidated and could provide further therapeutic targets.

## Methods

### BMSC isolation and culture

Human primary bone marrow mesenchymal stromal cells (BMSC) were isolated from marrow aspirates. The aspirates were obtained from the iliac crest of 10 healthy donors (7 males and 3 females, ranging in age from 22 to 48 years old) during routine orthopaedic surgical procedures according to established protocols, after written informed consent by the patients. The methods were performed in accordance with relevant guidelines and regulations and approved by the local ethical committee Ethik Kommission Beider Basel (Ref. 78/07). Cells were isolated and cultured as previously described^19,39^. Briefly, after centrifugation, the cell pellet was washed in PBS (Gibco^TM^, Thermo Fisher Scientific, Waltham, Massachusetts, USA), resuspended in α-MEM medium (Gibco^TM^, Thermo Fisher Scientific, Waltham, Massachusetts, USA) containing 10% bovine serum (HyClone, South Logan, Utah, USA), 1 mM Sodium Pyruvate (Gibco^TM^, Thermo Fisher Scientific, Waltham, Massachusetts, USA), 10 mM HEPES Buffer Solution (Gibco^TM^, Thermo Fisher Scientific, Waltham, Massachusetts, USA) and 5 ng/ml FGF-2 (R&D System Minneapolis, Minnesota, USA) and plated at a density of 10^5^ nucleated cells/cm^2^. BMSC were cultured in 5% CO^2^ at 37 °C.

### Recombinant TG-VEGF production and purification

The engineered cross-linkable form of mouse VEGF-A 164 was produced as previously described^19^. Briefly, before insertion into the expression vector pRSET (Invitrogen, Carlsbad, California, USA), the cDNA for mouse VEGF-A 164 was amplified by PCR using primers designed to add the transglutaminase substrate sequence NQEQVSPL, including the 8 N-terminal residues of α2-plasmin inhibitor (α2PI 1–8), onto the N-terminus of the amplified cDNA. The engineered protein was expressed in Escherichia coli strain BL21 (D ε3) pLys (Novagen, Merck, Darmstadt, Germany). The recombinant α2 PI 1–8 -VEGF-A 164 (TG-VEGF) was isolated from inclusion bodies, processed and refolded following a modified version of a previously published protocol^40^. Briefly, the bacteria were lysed in triton X-100 by sonication and the inclusion bodies were collected from the lysate by centrifuging. A washing with Tri- ton X114 was used to remove membrane proteins and endotoxins. Proteins were extracted with urea buffer overnight at 4 °C under magnetic stirring. Urea concentration was slowly reduced by dialysis and further dimerization of TG-VEGF was obtained with a redox system (0.5 mM oxidized glutathione, 5 mM reduced glutathione) under stirring for 48 h at 4 °C. After, 24 h dialysis against Tris- buffered saline (TBS) was used to remove glutathione and urea. Proteins were then concentrated using a 10-kDa Amicon tube (Millipore, Merck, Darmstadt, Germany) and further filtered with 0.22-μm filters. TG-VEGF monomers and dimers were separated using size exclusion with a HiLoad 16/60 Superdex 75-pg column (GE healthcare, Chicago, Illinois, USA). Fractions corresponding to TG-VEGF dimers were pooled together, concentrated with Amicon tubes, and filtered through a 0.22-μm filter. SDS/PAGE was used to assess TG-VEGF dimers purity (>99%). Endotoxin level was verified to be under 0.05 EU/mg of protein using the human embryonic kidney (HEK)-Blue mTLR4 assay (Invivogen, San Diego, California, USA).

### Recombinant TG-Aprotinin production and purification

The engineered cross-linkable form of aprotinin was produced as previously described^19^. Briefly, the cDNA for bovine aprotinin was modified at its N-terminus to add an 6x histidine tag and a thrombin cleavage site, and at its C-terminus with the cDNA of the transglutaminase substrate sequence NQEQVSPL. The cDNA was then subcloned into a pXLG vector for expression in HEK-293F mammalian cells. HEK-293F cells were transiently transfected using polyethylenimine (PEI) and cultured in suspension for 7 days, after what the supernatant was collected and purified via immobilized metal affinity chromatography (HisTrap columns, GE healthcare, Chicago, Illinois, USA) using an Akta Pure FPLC system (GE healthcare, Chicago, Illinois, USA). The purified proteins were then dialyzed and the histidine tag was cleaved using thrombin (50 U/mg) for 24 h at room temperature. The proteins were purified again using HisTrap and benzamidine columns (GE healthcare, Chicago, Illinois, USA) to remove the cleaved his-tag and thrombin. The purified TG-aprotinin was dialyzed against TBS, concentrated using Amicon tubes, filtered through a 0.22-μm filter and stored at −80 °C.

### Generation and in vivo implantation of osteogenic constructs

60 mm^3^ of silicate-substituted calcium phosphate granules of 1–2 mm size (Actifuse®; Apatech-Baxter, Elstree, UK) where mixed with 1 × 10^6^ BMSC at first passage after initial confluence (P1) and embedded in a 60-µl fibrin gel prepared by mixing 25 mg/ml human fibrinogen (plasminogen-, von Willebrand Factor-, and fibronectin-depleted; Milan Analytica AG, Rheinfelden, Switzerland), 3 U/mL factor XIII (CSL Behring, King of Prussia, Pennsylvania, USA), and 6 U/ml thrombin (Sigma-Aldrich, St. Louis, Missouri, USA) with 2.5 mM Ca^2+^ in 4-(2-hydroxyethyl)-1- piperazineethanesulfonic acid (Hepes, Lonza, Basel, Switzerland). The final constructs were roughly spherical with a diameter of about 6 mm. Fibrin gels decorated with 51 µg/ml of aprotinin-α2PI_1–8_ and 0.01, 0.1, 1, 10 or 100 µg/ml of α2PI_1–8_-VEGF-A_164_ were obtained by adding the engineered proteins to the cross-linking enzymes solution before mixing with fibrinogen. Osteogenic grafts were allowed to polymerize at 37 °C for 10 min after mixing before in vivo implantation. The resulting constructs were implanted subcutaneously (4 constructs/animal) in 5-weeks old female nude mice (CD1-*Foxn1*^*nu*^, Charles-River, Sulzfeld, Germany). Studies were performed in age- and sex-matched young animals in order to reduce sources of variability in the efficiency of bone formation, and not to investigate the influence of age and gender. Animals were treated in agreement with Swiss legislation and according to a protocol approved by the Veterinary Office of Canton Basel-Stadt (permission #1797). Four to ten constructs were implanted for each condition (*n* = 4–10 samples/group), generated with cells from 5 independent donors (at least 2 replicates/donor) and in a minimum of 2 independent experiments per condition. After 1, 4 and 8 weeks, mice were sacrificed by inhalation of CO_2_ and constructs were explanted.

### Histological processing and immunofluorescence tissue staining

Explanted constructs were washed with PBS and fixed overnight at +4 °C with freshly prepared 1% paraformaldehyde (Sigma-Aldrich, St. Louis, Missouri, USA) in PBS. Subsequently, the samples were decalcified in a PBS-based solution containing 7% w/v EDTA (0.5 M, pH 8, Sigma-Aldrich, St. Louis, Missouri, USA) and 10% w/v sucrose (Sigma-Aldrich, St. Louis, Missouri, USA) and incubated at 37 °C on an orbital shaker. The solution was renewed daily for about 20 days, until the samples were fully decalcified, as estimated by the degree of sample stiffness. Finally, the samples were embedded in OCT compound (CellPath LTD, Newtown, UK), frozen in freezing 2-methylbutane (isopentane) (Sigma-Aldrich, St. Louis, Missouri, USA) and 10 µm-thick sections were obtained with a cryostat.

Immunofluorescence staining was performed with the following primary antibodies and dilutions: rat anti-mouse CD31 (clone MEC 13.3, BD Bioscience, San Jose, California, USA) at 1:100; mouse anti-Human nuclei (clone 235-1, Merk Millipore, Darmstadt, Germany) at 1:200; rabbit anti-Ki67 (Abcam, Cambridge, UK) at 1:100; rabbit anti-Cleaved Caspase 3 (Asp175; Cell Signaling Technology, Danvers, Massachusetts, USA) at 1:200; mouse anti-human BSPII (Clone LFMb-24, Santa Cruz Biotechnology, California, USA) at 1:50; rabbit anti-Osterix (SP7; Abcam, Cambride, UK) at 1:200; rabbit anti-cleaved NICD1 (Cell Signaling Technology, Danvers, Massachusetts, USA) at 1:100. Fluorescently labeled secondary antibodies (Invitrogen, Thermo Fisher Scientific, Waltham, Massachusetts, USA) were used at 1:200.

Fluorescence images were acquired with an Olympus BX63 (Olympus, Münster, Germany) and a Nikon Ti2 Eclipse microscope (Nikon, Tokyo, Japan). All image measurements were performed with cellSens software (Olympus, Münster, Germany), NIS-Elements (Nikon, Tokyo, Japan) and FIJI software (ImageJ, http://fiji.sc/Fiji).

All subsequent quantifications were performed by 3 independent observers who were blinded to the sample identities.

### Angiogenesis

Invasion of osteogenic constructs by blood vessels, as well as vascular density were assessed after 1 week in vivo by immunostaining for CD31. Complete images of whole sections from the central part of each sample were acquired (*n* = 6 samples/group) and the area of invasion was measured by tracing the area occupied by blood vessels (CD31^+^ structures) and expressed as percentage of the total graft area. To quantify vessel density at week 1, at least 15 images were acquired per sample within the invaded areas (*n* = 6 samples/group) and vessel length density (VLD) was measured tracing the total length of vessels in the fields and by normalizing it to the field area (mm/mm^2^). Total vessels length (mm) was obtained multiplying the measured VLD by the area invaded by blood vessels. VLD at 4 and 8 weeks was quantified on 15 randomly acquired images, covering all the area of the tissue section, since constructs were completely invaded by these time-points.

### Proliferation and apoptosis

Proliferation and apoptosis of implanted human progenitor cells were quantified after 1 week in vivo by immunostaining for Ki67 or Cleaved-Caspase3, respectively, together with anti-Human nuclei. Images of whole sections for each condition (*n* = 6 samples/group) were divided in three concentric layers, each spanning a depth of 500 µm from the external surface, and the remaining central part was considered the core (Fig. 3a). Ki67^+^ or Caspase3^+^ human cells were manually counted in 6–8 fields of 300 µm^2^-area within each layer and expressed as percentage of the total number of human cells in the field.

### Bone formation

Bone tissue was detected by Masson’s trichrome staining (Réactifs RAL, Martillac, France), performed according to manufacturer’s instructions. Twenty whole-section reconstructions per sample (*n* = 6 samples/group) were acquired with transmitted light and bone tissue was quantified tracing the area occupied by mineralized matrix (dark green staining) and normalizing it by the total area of the section. In addition, the presence of mature bone (red staining) matrix was measured and normalized by the total amount of bone.

### Osteoclast detection

In order to detect osteoclasts, sections were stained for tartrate-resistant acid phosphatase (TRAP) activity. Briefly, after rinsing with water, slides were incubated for 20 min with 0.1 M Acetate Buffer (0.2 M Sodium Acetate, 0.2 M Acetic Acid, 50 mM Sodium L-tartrate dibasic dihydrate, pH 5.0) and then stained with 1 mg/ml of Fast Red LB salt (Sigma-Aldrich, St. Louis, Missouri, USA) and 1 mg/ml of naphtol AS-MX phosphate (Sigma-Aldrich, St. Louis, Missouri, USA) dissolved in 0.1 M acetate buffer for 1 h at 37 °C. After TRAP staining, nuclear counter staining was performed with Haematoxylin for 1 min at room temperature. TRAP-positive cells were quantified on 15 randomly-chosen fields per construct in 6 constructs/condition (*n* = 6 samples/group). Multinucleated TRAP + cells in the fields were counted manually and the total number was normalized by the field area.

### Quantification of osterix (OSX) positive human cells

The number of osteogenic committed human cells was assessed after 1 and 4 weeks in vivo by immunostaining for OSX in combination with an anti-human nuclei antibody (HuNu) and a blue-fluorescent DNA dye (DAPI). For the first week time point, images of whole sections for each condition (*n* = 5 samples/group) were acquired. A concentric layer of 1 mm depth from the external surface was traced and the OSX- positive human cells were automatically detected and counted using a custom-made macro for FIJI Software. Briefly, a region of interest (ROI) was traced within the 1 mm layer, thresholding was applied to the nuclei (DAPI), human nuclei and OSX channel; the number of OSX + human cells was obtained by colocalization of the three individual channels and expressed as percentage of the total number of human cells present inside the ROI.

Quantification of the number OSX- positive human cells at 4 weeks was performed as described above but on 10 randomly acquired fields per conditions throughout the entire construct (*n* = 6 samples/group).

### Quantification of bone sialoprotein II (BSPII)

To quantify the amount of human BSP, immunofluorescence staining was performed and 5–7 random fields (*n* = 6 samples/group) were acquired per each condition. The amount of human BSP was quantified using a custom-made macro in FIJI software. Briefly, a region of interest (ROI) was traced, thresholding was applied to the human BSP channel and the number of pixels above the threshold was normalized by the total number of the pixels of the ROI. The number of human cells (detected by immunofluorescence staining for an anti-human nuclei antibody) was quantified automatically on a whole section per each sample (*n* = 6 samples/group) using FIJI software and normalized by the tissue area.

### Quantitative real-time PCR

For RNA extraction from osteogenic grafts, constructs were immediately frozen in liquid nitrogen after harvesting (*n* = 8–10 samples/group). Tissues were disrupted and homogenized using a Qiagen Tissue Lyser (Qiagen, Basel, Switzerland) in 1 ml TRIzol Reagent (Thermo Fisher Scientific, Waltham, Massachusetts, USA) for every 100 mg of tissue. Total RNA from lysed tissues was isolated with a RiboPure RNA purification kit (Thermo Fisher Scientific, Waltham, Massachusetts, USA) according to manufacturer’s instruction. Total RNA from in vitro-cultured human BMSC (*n* = 6, from 3 independent donor) was isolated with a Quick-RNA Miniprep plus kit (Zymo Research Europe GbmH, Freiburg im Breisgau, Germany) according to manufacturer’s instruction.

RNA from tissues and human BMSC was reverse-transcribed into cDNA with the SuperScript III Reverse Transcriptase (Thermo Fisher Scientific, Waltham, Massachusetts, USA). Quantitative Real-Time PCR (qRT-PCR) was performed on an ABI 7300 Real-Time PCR system (Applied Biosystems, Foster City, California, USA). Expression of genes of interest was determined using the following human-specific TaqMan gene expression assays (Thermo Fisher Scientific, Waltham, Massachusetts, USA): Runx2 (Hs01047973_m1); SP7/Osterix (Hs01866874_s1); BSP (Hs00913377_m1); BGLAP/Osteocalcin (Hs01587814_g1). Reactions were performed in duplicate for each template, and normalized to expression of the GAPDH housekeeping gene (Hs02786624_g1). Relative mRNA expression was defined as 2^−ΔCt^, where _Δ_Ct = Ct Target Gene – Ct GAPDH. The significance of differences was calculated on _Δ_Ct values.

### Statistics

Data are presented as mean ± standard error of the mean (SEM). The significance of differences was assessed with the GraphPad Prism 9 software (GraphPad Software, San Diego, California, USA). The normal distribution of all data sets was tested and, depending on the results, multiple comparisons were performed with the parametric one‐way analysis of variance (ANOVA) followed by the Bonferroni test, or with the nonparametric Kruskal–Wallis test followed by Dunn’s post‐test. Percentage of proliferating and dying human cells were first normalized by log2‐transformation and then analyzed by one‐way ANOVA followed by Bonferroni test for multiple comparisons. Differences were considered statistically significant if *p* < 0.05.

## Data Availability

The datasets generated and/or analysed during the current study are available from the corresponding author on reasonable request.
